# The Use of Tuberculin

**Published:** 1904-10-15

**Authors:** 


### THE USE OF TUBERCULIN.

Dr. Albert S. F. Grünbaum [1] is of opinion that the neglect of tuberculin as a therapeutic agent is a concession to popular prejudice rather than the result of unfavourable experience. The evidence, he considers, warrants the conclusion that in suitable cases the remedy has specific value, and to omit it in these instances is to assume a grave responsibility. In his own experience Dr. Grünbaum has not seen any other form of treatment produce results approaching those following the use of tuberculin. He employs the neu-tuberculin (bacilli emulsion) of Koch, and avoids the production of violent reaction. During the earlier part of the treatment the patient is kept in hospital, but when the dose has reached 0·75 cubic centimetre (beyond which it is rarely necessary to go) one injection is given every week in the out-patient room, the patient remaining at home on the following day. In no circumstances is an injection given until clinical recovery from the effects of the preceding one has been attained. The treatment must extend over a period of three to six months. The method has its failures, like other modes of treatment, but probably not so many, other things being equal, as the sanatorium. If possible the use of tuberculin should be combined with residence at a sanatorium, but even without the latter the injections have distinct value. It has been shown by Dr. A. E. Wright that tuberculin acts as a stimulus on the cells of the body, and so leads to an increase in the protective substances which these cells produce. Dr. Grünbaum also suggests that now the effects of tuberculin can be gauged it should be used as a prophylactic, especially in cases of obvious tuberculous diathesis. Dr. W. Camac Wilkinson [2] also holds that tuberculin is a specific remedy in tuberculosis, but insists that if other micro-organisms are associated with the tubercle bacilli in the morbid process, tuberculin may fail, or may even prove harmful. The failure of 1891 was due, he suggests, to a disregard of the limitations and restrictions laid down by Koch, and to a general ignorance of the *rôle* of mixed infection in pulmonary tuberculosis. The recognition of this *rôle* is an indispensable condition of success in the use of tuberculin, and it can only be appreciated by the aid of thorough and careful bacteriological examination of the sputa. Dr. Wilkinson has used tuberculin R. (TR) chiefly, but also the old or original preparation. After watching the effects week by week for five years in 70 cases he is emphatic in favour of the treatment provided there is no mixed infection. In cases in the first stage permanent improvement is to be expected, the bacilli disappearing from the sputa, and ultimately the sputa ceasing altogether. In the later stages of the disease, as is to be expected, the results are less satisfactory, but even here in many cases considerable improvement may be obtained. In no instance in Dr. Wilkinson's experience did tuberculin exercise any prejudicial action and he has never found it rouse into activity a latent tubercular focus. In many of his cases the patient hardly lost a day's work during the course of the treatment and proof of the care with which the tuberculin is made is afforded by the fact that not one of all the thousands of injections was followed by a skin abscess or other local accident.

[1] Lancet, September 24th, 1904. [2] Brit. Med. Journ., June 7th, 1902.

